# Pherotype Influences Biofilm Growth and Recombination in *Streptococcus pneumoniae*

**DOI:** 10.1371/journal.pone.0092138

**Published:** 2014-03-19

**Authors:** Margarida Carrolo, Francisco Rodrigues Pinto, José Melo-Cristino, Mário Ramirez

**Affiliations:** 1 Instituto de Microbiologia, Faculdade de Medicina, Universidade de Lisboa, Lisboa, Portugal; 2 Instituto de Medicina Molecular, Faculdade de Medicina, Universidade de Lisboa, Lisboa, Portugal; 3 Centro de Química e Bioquímica, Faculdade de Ciências, Universidade de Lisboa, Lisboa, Portugal; Instituto Butantan, Brazil

## Abstract

In *Streptococcus pneumoniae* the competence-stimulating peptide (CSP), encoded by the *comC* gene, controls competence development and influences biofilm growth. We explored the influence of pherotype, defined by the two major *comC* allelic variants (*comC1* and *comC2*), on biofilm development and recombination efficiency. Among isolates recovered from human infections those presenting *comC1* show a higher capacity to form *in vitro* biofilms. The influence of pherotype on biofilm growth was confirmed by experiments with isogenic strains differing in their *comC* alleles. Biofilm architecture evaluated by confocal laser scanning microscopy showed that strains carrying *comC1* form biofilms that are denser and thicker than those carrying the *comC2* allele. Isogenic strains carrying the *comC1* allele yielded more transformants than those carrying the *comC2* allele in both planktonic and biofilm growth. Transformation assays with *comC* knockout strains show that ComD1 needs lower doses of the signaling peptide to reach the same biological outcomes. In contrast to mixed planktonic growth, within mixed biofilms inter-pherotype genetic exchange is less frequent than that occurring between bacteria of the same pherotype. Since biofilms are a major bacterial lifestyle, these observations may explain the genetic differentiation between populations with different pherotypes reported previously. Considering that biofilms have been associated with colonization our results suggest that strains carrying the *comC1* allele may be more transmissible and more efficient at persisting in carriage. Both effects may help explain the higher prevalence of the *comC1* allele in the pneumococcal population.

## Introduction

Although planktonic bacteria are the most frequently studied microbial lifestyle *in vitro*, it is comparatively rare *in vivo*, where most prokaryotes grow in matrix-enclosed biofilms. Life within a biofilm matrix, wherein oxygen is limited and the metabolic rate is altered, results in several benefits, including protection from environmental threats such as host immune defenses, antibiotics, and surfactants [1]–[3]. These biological structures have also been proposed as the way bacteria organize themselves when colonizing asymptomatically the upper airways of their human hosts [4]–[6].

Bacteria are known to communicate with each other within biofilms in order to set up mutually beneficial associations and coordinate protection measures. Communication flows through quorum sensing system (QS), where diffusible chemical signals (autoinducers) interact with specific receptors on the surface or in the cytoplasm of bacteria, regulating the expression of specific target genes [7].

Induction of the competent state in streptococci belonging to the mitis phylogenetic group is controlled by the extracellular concentration of a secreted peptide pheromone, called the competence-stimulating peptide (CSP) the processed product of the *comC* gene [7]–[9]. In several streptococci, the functioning of the QS system depends on the products of two distinct genetic loci, *comAB* and *comCDE* [8], [10]. The operon *comAB* encodes the secretion apparatus necessary for CSP maturation and export whereas the *comCDE* operon encodes the immature signal (ComC) and the necessary sensing machinery. The signaling cascade, which leads to the induction of competence, begins with the interaction of CSP with its cognate receptor, ComD. When CSP reaches a critical concentration it binds to the membrane-bound histidine kinase receptor ComD, causing its autophosphorilation. Phosphorylated ComD transfers the phosphate group to an intracellular response regulator ComE, which then upregulates the transcription of the *comAB* and *comCDE* operons. Phosphorylated ComE also drives the expression of *comX* that encodes an alternative sigma factor, so that in response to the signal-peptide, not only more CSP is produced but a relatively large number of genes are induced (aprox. 6% of total genome) [11]. Only a small part of these genes have direct roles in DNA uptake, suggesting that the QS system of *S. pneumoniae* plays a larger role in the biology of this species. A set of genes induced at competence functions to trigger the lysis of a fraction of the bacteria within a population [10], [12], [13]. This process of “fratricide” has been postulated to be an important source of DNA for competent cells.

Several allelic variants of the *comC* gene have been identified in *S. pneumoniae*, although the vast majority of isolates carries one of two variants: *comC1* or *comC2* resulting in the production of two distinct peptides CSP1 and CSP2 [14]–[16]. Each different CSP variant identifies a pherotype and strains genetically carrying one of the variants are mostly unable to respond to the presence of the other signaling peptide, possibly due to receptor specificity [14], [15].

The existence of two predominant pherotypes in *S. pneumoniae* and the documented occurrence of co-colonization [17]–[19], led to the proposal of two contrasting models regarding the pherotype impact on genetic exchange [10]. In the first model, absence of inter-pherotype cross-activation favors the induced lysis of bacteria belonging to the other pherotype [20]. Consequently, DNA of bacteria not sharing the same pherotype would be released and become available for transformation, resulting in higher genetic diversity within the pneumococcal population [21]. The second model is based on the premise that the lack of inter-pherotype communication prevents genetic exchange and favors differentiation [22], [23]. Recently, we have reported that the two predominant pherotypes define genetically differentiated subpopulations of pneumococci consistent with the existence of limitations to gene flow between different pherotypes. We proposed that this “assortative mating” mediated by the pherotypes may be driving genetic diversification within *S. pneumoniae* [16].

In streptococci, several reports have shown a link between CSP production and biofilm formation [7], [24]–[27]. In *S. gordonii* a mutant where *comD* was inactivated presented impaired biofilm growth [27]. Further evidence comes from a report in *S. mutans* where the inactivation of any component of the *comCDE* operon resulted in a phenotype of defective competence and biofilm formation [26]. An alternative approach showed that the addition of synthetic CSP favored the biofilm mode of growth of *S. intermedius* without affecting the rate of culture growth [25]. A previous study in *S. pneumoniae* supports the link between competence and biofilm formation showing that CSP receptor mutants are incapable of forming biofilms [28]. We have recently shown that spontaneous phage-mediated lysis promotes pneumococcal biofilm formation through the release of eDNA [16] and competence induced fratricide is also an important mechanism contributing to the accumulation of this important biofilm matrix component [29].

Given the described links between CSP signaling, biofilm growth and genetic recombination, we pursued the hypothesis that different pherotypes could vary in their capacity for biofilm growth, and that these differences could influence the recombination efficiency between strains with different pherotypes. Oggioni and colleagues had already reported some preliminary data showing that strains carrying the *comC1* allele had higher numbers of cells attached to a plastic surface [28]. Here we show that among strains recovered from human infections, those carrying the *comC1* allele are better biofilm producers, an observation that was corroborated with isogenic strains differing only in their pherotypes. In contrast to what happens in planktonic growth, when strains of different pherotypes are grown together in biofilms, intra-pherotype genetic transfer is favored over intra-pherotype exchanges. These observations may have important consequences for the evolution of *S. pneumoniae*.

## Materials and Methods

### Bacterial strains and growth conditions

The 90 invasive pneumococcal isolates were recovered in Portugal during the period of 1999 to 2002 and are part of the Faculdade de Medicina de Lisboa collection. Bacterial strains R36A and TIGR4 were obtained from the Rockefeller University collection (A. Tomasz). Strain CP1500, which carries resistance genes for both streptomycin and novobiocin [30], was used as a source of DNA for transformation assays. All *S. pneumoniae* strains were grown in a casein-based semi-synthetic medium (C+Y) at 37°C without aeration [31] or in tryptic soy agar (TSA) (Oxoid, Hampshire, England) supplemented with 5% (v/v) sterile sheep blood incubated at 37°C in 5% CO_2_.

### Liquid medium growth curves

An inoculum of 50 μl of rapidly thawed bacteria was added to a glass tube with C+Y to reach a total volume of 5 ml. Bacteria were incubated at 37°C for 340 minutes and growth was monitored by OD_600nm_ every 30 minutes. For each strain, 3 replicates of 3 independent experiments were obtained.

### Pherotype detection by PCR

The *comC1* (CSP1) and *comC2* (CSP2) gene fragments were amplified as previously described [16]. Briefly, a multiplex PCR with primers CSP_up (5′-TGA AAA ACA CAG TTA AAT TGG AAC-3′), CSP1_dn (5′-TCA AGA AAG GAT AAA GGT AGT CCT C-3′) and CSP2 _dn (5′-TAA AAA TCT TTC AAT CCC TAT TT-3′) was used to amplify fragments of 620 bp for the *comC1* allele and 340 bp for the *comC2* allele. Template DNA was prepared by diluting 9 μl of an overnight culture in 441 μl of water and boiling this mixture for 2 minutes. The multiplex PCR reactions were performed in 50 μl of final volume containing 20 μl of template solution, 1× reaction buffer (Biotools, Madrid, Spain), 10 mM dNTPs (Fermentas, Vilnius, Lithuania), 20 pmol of each of the primers and 1.25 U GoTaq Polymerase (Invitrogen, Carlsbad, California). The PCR program consisted of 30 cycles of denaturation at 95°C for 1′, annealing at 55°C for 30″, primer extension at 72°C for 1′ and a final 10′ incubation at 72°C to complete extension.

### Altered pherotype strain construction

To construct strain R36A-CSP2, the operon *comCDE* of TIGR4 strain (CSP2 pherotype) was amplified by PCR reaction. Likewise, construction of strain TIGR4-CSP1 started with the amplification of the operon *ComCDE* of R36A (CSP1 pherotype). In both cases, primers ComCDE_fw (5′-GCTGACCGTGATTTCGTTATTGTG-3′) and ComCDE_rev (5′-AGGATAAGTATGATATGATTGAGC-3′), were used allowing the amplification of fragments with 3415 bp encompassing the entire *comCDE* operon. Template DNAs were prepared by diluting 9 μl of an overnight culture in 441 μl of water and boiling this mixture for 2′. In both cases, the PCR reaction was performed in 50 μl of final volume containing 20 μl of template DNA solution, 1× reaction buffer (Invitrogen, Carlsbad, California), 10 mM of dNTPs (Invitrogen, Carlsbad, California), 2 mM of MgCl_2_ (Invitrogen, Carlsbad, California), 0.4 pmol of each of the primers and 1.25 U GoTaq Polymerase (Invitrogen, Carlsbad, California). The PCR program consisted of an initial 10 cycles of 10″ denaturation at 94°C, 30″ of annealing at 53°C and 30″ of primer extension at 68°C. A second run of 10 cycles started with 15″ of denaturation at 94°C, 30″ of annealing at 53°C and primer extension of 2′ 30″ at 68°C, with a 20″ increase in each successive cycle. The amplification ends with 7′ incubation at 68°C to complete extension. The resulting fragments were purified with the High Pure PCR Product Purification kit (Roche Applied Sciences, Germany) and used directly for transformation with R36A or TIGR4, as previously described [32], briefly a 5 ml culture of each strain was grown to an optical density at 600 nm of 0.06 and inducing development of competence with CaCl_2_ (0.5 mM), bovine serum albumin (0.002%) and CSP1 (250 ng/ml) in case of strain R36A or CSP2 (250 ng/ml) for TIGR4. After 5 minutes, 1 ml of induced cells were incubated with 100 ng of donor DNA for 70 min. To select R36A-CSP2 and TIGR4-CSP1 mutants, colony pooling was performed using PCR for pherotype identification.

### Antibiotic resistant strains

The following antibiotic resistant strains were constructed: R36A(NovR) (novobiocin resistant, streptomycin sensitive), R36A(StrepR) (streptomycin resistant, novobiocin sensitive), R36A-CSP2(NovR) (novobiocin resistant, streptomycin sensitive), R36A-CSP2(StrepR) (streptomycin resistant, novobiocin sensitive). These strains were constructed by transforming R36A and R36A-CSP2 with genomic DNA from the novobiocin and streptomycin resistant strain CP1500. Transformants were selected by plating on tryptic soy agar (Oxoid, Basingstoke, United Kingdom) supplemented with 5% sheep blood (blood agar), containing 5 μg/ml of novobiocin or 100 μg/ml of streptomycin. Transformants resistant to one antibiotic were tested for growth on blood agar with the other antibiotic. Only transformants that presented resistance to one of the antibiotics were selected. Antibiotic resistant strains TIGR4 and TIGR4-CSP1 were constructed similarly.

### Biofilm growth and quantification

Biofilm formation was determined in 96-well flat-bottom polystyrene microtiter dishes (Nunc™, Roskilde, Denmark). Cells were grown in C+Y medium to an OD600nm between 0.5 and 0.6 and then diluted 1∶4 to a final volume of 200 μl per well. Plates were incubated at 37°C and biofilm mass was determined using the crystal violet staining protocol [33]. Biofilm mass was measured at OD595nm using a plate reader (Bio-Rad Model 680, Microplate Manager™ software V5.2.1). For the evaluation of viable cells in biofilms, a previously described procedure was adopted [34]. Biofilms were grown in 96-well plates for 24 h at 37°C in 5% CO2. Culture medium was aspirated from the wells to remove planktonic bacteria. Wells were washed with isotonic phosphate buffer (0.15 M, pH 7.2) twice. Biofilms were scraped thoroughly from each well and well contents were aspirated and placed in 1 ml of isotonic phosphate buffer. Total CFU number was determined by serial dilution and plating on appropriate media.

For studying the impact of CSP on biofilm formation, CSP1 (NH2-EMRLSKFFRDFILQRKK-COOH) and CSP2 (NH2-EMRISRIILDFLFLRKK-COOH) (CASLO Laboratory, Lyngby, Denmark) were added to the biofilm growth medium at t = 0. Biofilm mass quantification was performed at t = 24 h corresponding to maximal biofilm growth as determined previously [16]. The purity of the synthetic peptides was evaluated by mass spectrometry and high-performance liquid chromatography and determined to be >98% for both peptides.

### Transformation within and between strains of different pherotypes

For the biofilm model, an equal number of isogenic *comC1* and *comC2* bacteria, each strain carrying a different antibiotic resistance marker, was seeded in 96-well plates and after 24 h of biofilm growth, transformants within these mixed strain biofilms were evaluated. For evaluating transformation in the liquid culture, isogenic *comC1* and *comC2* strains, each carrying a different antibiotic resistance marker, were individually cultured until OD_600nm_ = 0.3 was reached. Equal volumes of both cultures were mixed, diluted three fold and incubated until OD_600nm_ = 0.8. In both biofilm and liquid culture, similar mixtures of two strains with the same pherotypes but different antibiotic resistance markers were used as control. The proportion of doubly resistant CFUs was indicative of transformation.

### Construction of the mutant strains Δ*comC1* and Δ*comC2*

The chloramphenicol acetyltransferase (CAT) gene, which confers resistance to the antibiotic chloramphenicol was used as a selectable-marker in the construction of the mutants: R36A Δ*comC*, R36A-CSP2 Δ*comC*, TIGR4 Δ*comC*, TIGR4-CSP1 Δ*comC*. The gene was amplified from the PVP3 plasmid using the set of primers BM18, fw (GGGTTCCGAGGCTCAACGTCAA) and PVP3-1R, rev(CGAGGTCGACGGTATCGATAAGCT), resulting in a 1053 bp fragment. The PCR reaction was performed in 50 μl of final volume containing 10 μl of template DNA solution, 10× reaction buffer (Invitrogen, Carlsbad, California), 10 mM of dNTPs (Invitrogen, Carlsbad, California), 2 mM of MgCl_2_ (Invitrogen, Carlsbad, California), 0,4 pmol of each of the primers and 1.25 U of High Fidelity Polymerase (Invitrogen, Carlsbad, California). The PCR program consisted of a run of 30 cycles started with 1′ of denaturation at 94°C, 1′ of annealing at 58°C and primer extension of 1′ at 72°C, and a final 10′ amplification at 72°C to complete extension. The resulting fragments were purified with the High Pure PCR Product Purification kit (Roche Applied Sciences, Germany).

A region of ±500 bp immediately upstream the *comC* gene (fragment A) and another region, around the same size, immediately downstream the gene (fragment B) were amplified and linked to the CAT gene in order to obtain a construct that would substitute the *comC* gene in both a R36A (CSP1 background) and a TIGR4 (CSP2 background) strains yielding, respectively, Δ*comC1* and Δ*comC2*. Fragment A with 520 bp was amplified in the CSP1 background with primers fragAComC1_fw (CGTGATTTCGTTATTGTGTTAG) and fragAComC1_rev_CSP1 (TTGACGTTGAGCCTCGGAACCCACAACCTCATCTCCCCACC) with the first 22 nucleotides of this primer corresponding to the complementar of the BM18 primer. In the CSP2 background the set of primers used was fragAComC2_fw (CGTGATTTCGTTATTGTGTTAG) and fragAComC2_rev (TTGACGTTGAGCCTCGGAACCCAGATAAAATTCTCTCAACTGTCA) again with the complementar of BM18 included on this primer, resulting in a fragment of 480 bp. In the CSP1 background fragment B was amplified with primer fragBComC1_fw (AGCTTATCGATACCGTCGACCTCGTGAAATAAGGGGAAAGAGTAATGG) in which the first 24 nucleotides correspond to the reverse of the PEVP-1R primer and fragBComC1_rev (CGTCAAAATAGGCTAGTTCCAAATG), resulting in a 471 bp fragment. Fragment B in the CSP2 background was amplified with fragBComC2_fw(AGCTTATCGATACCGTCGACCTCGTGAAATAAGGAGAAAGAGTAATGG) also with the first 24 nucleotides corresponding to the reverse of the PEVP-1R primer and fragBComC2_rev (GGAGAGAGATAAAGACAATAGATG), resulting in a 618 bp fragment. All PCR reactions and programs for fragments A and B were similar to the ones used for the CAT gene amplification, with primer extension occuring at 52°C for fragment A and at 55°C for fragment B. All obtained fragments were purified with the High Pure PCR Product Purification kit (Roche Applied Sciences, Germany) prior usage. The next step involved the linkage of fragment A to CAT, both in CSP1 and CSP2 backgrounds. On these amplifications the following set of primers were used: fragA_ComC1_fw with PVP3-1R, resulting in a 1576 bp fragment and fragA_ComC2_fw with PVP3-1R, yielding a 1696 bp fragment. PCR reactions and programs were similar to the ones used in the CAT gene amplification, with primer extension occuring at 51°C for both amplifications. All fragments were purified as previously before usage. The following step included to link fragA-CAT to fragment B. In order to do so, a PCR was performed using the set of primers: fragAComC1_fw with fragBComC1_rv and fragAComC2_fw with fragBComC2_rv. PCR reactions and programs were similar to the ones used in the CAT gene amplification, with primer extension occuring at 53°C for both amplifications. The resulting fragments were used directly for transformation with R36A, R36A-CPS2, TIGR4 and TIGR4-CSP1, as previously described (37). Mutant strains were confirmed by sequence analysis.

### *ComD* efficiency assay

The transformation capacity of the mutant strains R36A Δ*comC*, R36A-CSP2 Δ*comC*, TIGR4 Δ*comC* and TIGR4-CSP1 Δ*comC* were tested in an assay with genomic DNA from the novobiocin and streptomycin resistant strain CP1500. In each case, the cognate CSP was added externally at the concentrations of 50, 100, 150, 200, 250, 300, 400, 600, 800 and 1000 ng/ml for CSP1 strains and of 200, 400, 600, 800, 1000, 1200, 1400, 1600, 1800, 2000 ng/ml for CSP2 strains. These values were selected after a preliminary assay and designed to have roughly the same number of points below and above the concentration that produces half the maximal transformation efficiency. Transformants were selected by plating on tryptic soy agar (Oxoid, Basingstoke, United Kingdom) supplemented with 5% sheep blood (blood agar), containing 100 μg/ml of streptomycin. Transformants resistant to this antibiotic were tested for growth on blood agar with the novobiocin antibiotic. Only transformants that presented resistance exclusively to streptomycin were considered.

### Confocal laser scanning microscopy

Biofilms were stained by using the Live/Dead BacLight kit (Invitrogen, Carlsbad, USA) and examined by confocal laser scanning microscopy. Images were acquired on a Zeiss LSM510 META confocal microscope (Carl Zeiss, Jena, Germany) using a PlanApochromat 63x/1.4 objective for cell viability assays and a C-AproChromat 40x/1.2. SYTO 9 fluorescence was detected using the 488 nm laser line of an Ar laser (45 mW nominal output) and a BP 505–550 filter. Propidium iodine fluorescence was detected using a DPSS 561 nm laser (15 mW nominal output) and a LP 575 filter. For imaging, the laser power was attenuated to 1–2% of its maximum value. The pinhole aperture was set to 1 Airy unit.

### Sequence analysis

To determine the similarity between the *comCDE* operon of strains R36A and TIGR4, an alignment of the amino acid sequences of the products of both operons was performed using the software Geneious Pro 4.0.4 (Biomatters, Auckland, New Zealand). The sequences with the following Genbank accession numbers were used: NC_003094 and NC_003028. To compare the promoter region of the operon *comCDE* of strains CSP1 (already described in [35]) and TIGR4 (CSP2), an alignment of nucleotide sequences was performed using the same software.

### Data analysis

One-way and two-way analysis of variance (ANOVA) were used to test effects of CSP level and pherotype on biofilm development or transformation efficiency, followed, when appropriate, by pairwise Tukey's post tests, using GraphPad Prism version 4.0a for Macintosh (GraphPad Software, San Diego, California USA). In the figures presented, error bars represent 95% Student's *t* confidence intervals for the sample mean. Non-linear least-squares regression was used to adjust the relationship between transformation efficiency and externally added CSP, using the nls function of the R statistical software (http://www.R-project.org/).

## Results

### Pherotype influences *in vitro* biofilm growth

**Figure 1:**
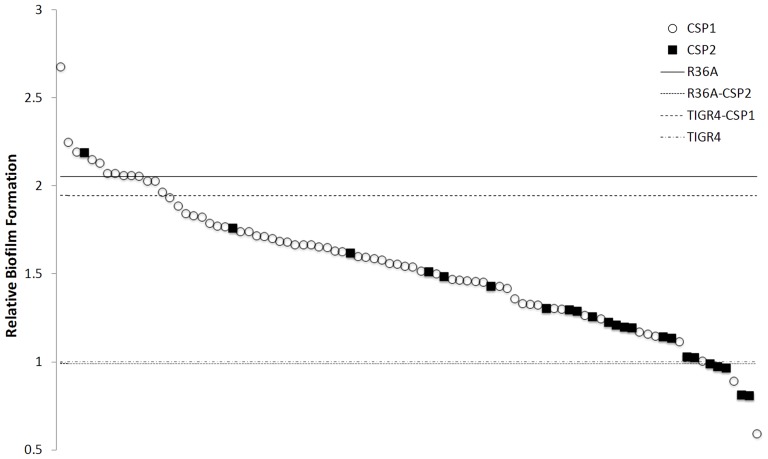
Distribution of pherotype-characterized strains according to the ability to form *in vitro* biofilms. A total of 90 strains with pherotype CSP1 (n = 67) or CSP2 (n = 23) were screened for their ability to form biofilms in 96-well plates without the addition of synthetic CSP. After 24h of incubation, the resulting biofilm was measured by crystal violet staining. Each plotted value is an average of 9 replicates normalized by the average biofilm mass measured for TIGR4 strain (OD_595nm_ = 0.147). Horizontal lines represent the relative biofilm formation of R36A, TIGR4 and their isogenic switched-pherotype mutant strains.

A total of 90 invasive pneumococcal isolates, representing 26 serotypes and 57 sequence types by multilocus sequence typing [36], [37] and screened for the *comC* allele carried by each isolate [16], were tested for their capacity to form *in vitro* biofilms in 96-well plates. Overall, isolates presenting the CSP2 pherotype showed a lower capacity to form biofilms (Figure 1). Since these strains represent diverse serotypes and genetic lineages this observation suggested that the pherotype impact on biofilm formation was independent of genetic background and other strain characteristics.

In order to better understand the influence of the two major pherotypes on biofilm growth, we constructed two strains where the pherotypes were switched relative to the ones presented by the wild-type (R36A-CSP2 and TIGR4-CSP1) with the objective of comparing their capacity to form biofilm with that of their otherwise isogenic wild-type strains. To do so, we extracted the operon *comCDE* of the donor strain (TIGR4 or R36A) and replaced it by recombination in the genome of the receptor strain. We also compared the DNA sequence of the previously described promoter region for operon *comCDE* in *comC1* strains [35] with the nucleotide sequence of a *comC2* strain and found that both promoter regions are identical, suggesting a similar regulation of expression of each of the pherotype variants.

The identity between the ComC, ComD and ComE of strains R36A and TIGR4 was determined by performing an alignment of the amino acid sequences of each protein. ComC includes 41 amino acids with an overall 81% identity between the two variants. However, all of the 8 differences are concentrated in its mature form (found in the extracellular medium), i.e. the 17 amino acid peptide that constitutes the CSP. The 441 amino acids of each of the ComD variants share 97% identity and ComE presents 250 amino acids that are 100% identical in the two strains. This indicates that the differences observed between the strains with the replaced pherotype and their isogenic counterparts must be caused by the alterations in ComC and ComD.

In liquid growth, the resulting strain R36A-CSP2 shows a similar generation time in the exponential growth phase (70.5 minutes; 95% confidence interval 68.4–72.6 minutes) than the wild-type strain R36A (69.3 minutes; 95% confidence interval 66.8-71.7minutes). The same pattern was observed with TIGR4 (81.4 minutes; 95% confidence interval 78.4–84.3 minutes) and TIGR4-CSP1 (79.0 minutes; 95% confidence interval 75.7–82.2 minutes).

**Figure 2:**
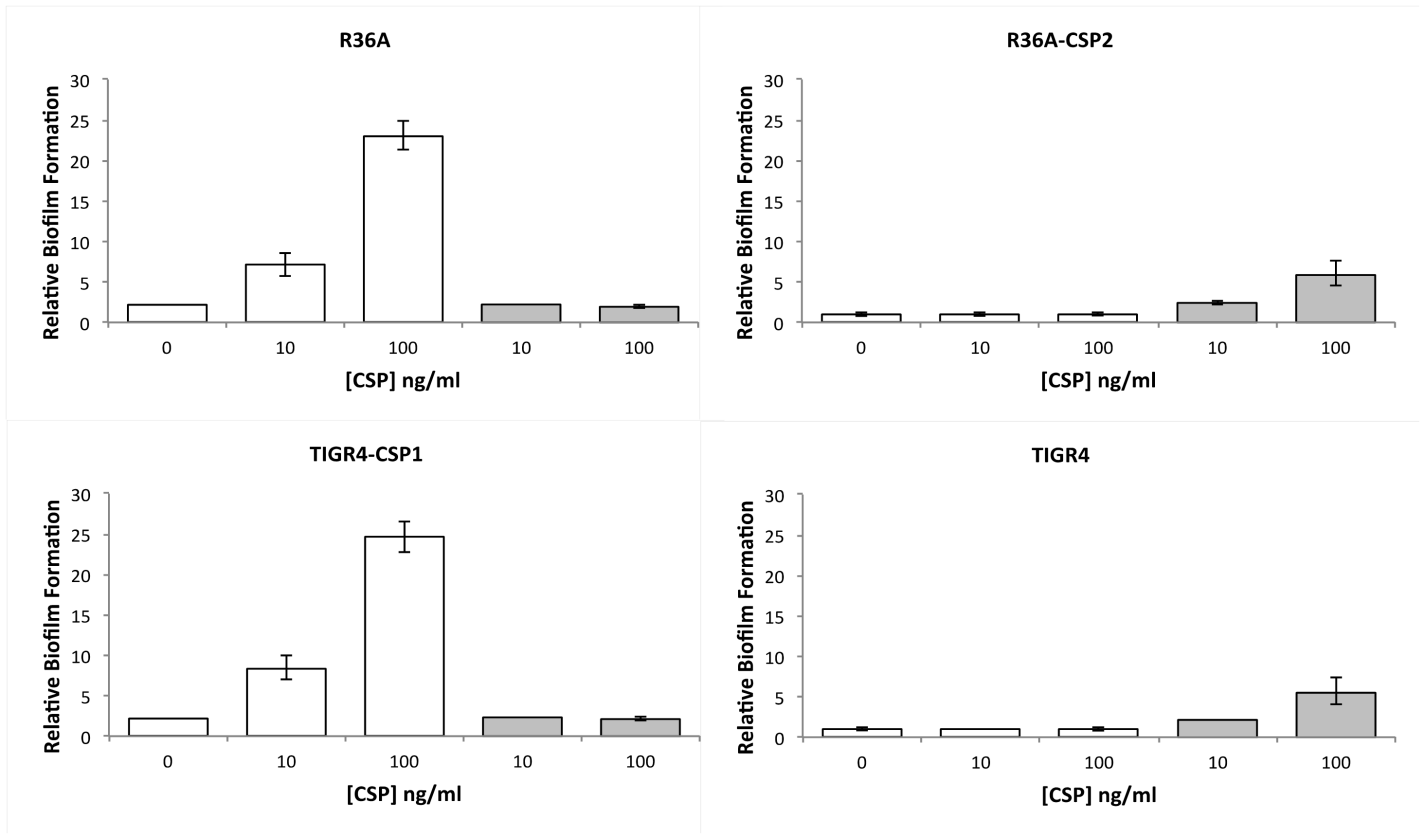
Biofilms of wild type and pherotype switched strains respond to cognate CSP in a dose-dependent manner. R36A and R36A-CSP2 and TIGR4-CSP1 and TIGR4 were incubated with synthetic CSP1 (white bars) and synthetic CSP2 (grey bars) during a biofilm growth experiment. Biofilm response to CSP was evaluated by determination of CFUs detached from the 96-well-plates after 24 h of incubation. Plotted values are relative to the average number of CFUs per well obtained for TIGR4 strain without adding CSP (2.0×10^8^ CFU/well). Error bars represent 95% confidence intervals for the mean of 3 independent experiments each with 3 technical replicates. Both strains were responsive to their cognate CSP but not to their heterologous CSP.

It had been previously shown that the addition of synthetic CSP to biofilms resulted in increased biofilm mass [28]. We decided to evaluate the capacity to form biofilms of both pherotype variants and of the wild-type strains. Additionally, we added synthetic CSP1 and CSP2 to each strain to verify if each responded exclusively to its cognate CSP. The impact on biofilm formation was measured by the determination of CFUs detached from the 96-well-plates after 24 h of incubation (Figure 2). Two-way ANOVA of the effects of the amount of cognate CSP and pherotype on biofilm viable counts shows that both factors have significant effects (p<10^−4^ in both cases) and there is also a significant interaction effect (p<10^−4^). The latter results from the amplification of the difference between pherotypes in the presence of cognate CSP. The same analysis applied to the effects of non-cognate CSP level and pherotype reveals that only the latter retains a significant effect, consistent with a lack of response to the non-cognate CSP. As expected, the results also show that R36A-CSP2 responds in a dose-dependent manner to synthetic CSP2 and not to CSP1 in contrast to the wild type and the reverse is true for TIGR4-CSP1. The specific sensing of each peptide (CSP1 and CSP2) by their cognate receptors was noted previously [14] and can be used as a functional assay to detect the pherotype of a given strain [15]. These results also indicate that isogenic CSP1 strains form better biofilms than their CSP2 counterparts, even if the strains have similar planktonic growth rates, and that this effect is amplified for the different synthetic CSP dosages used.

**Figure 3:**
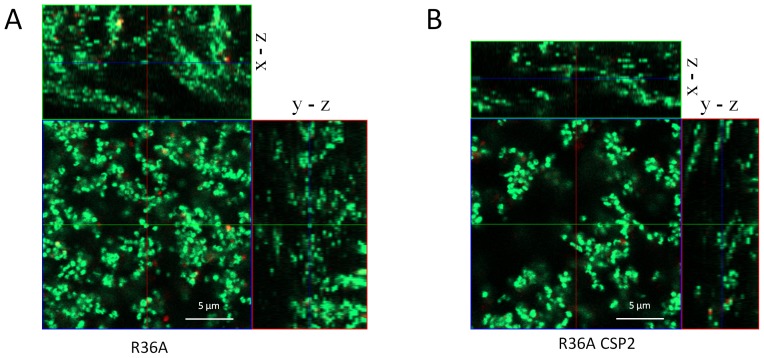
Confocal laser scanning microscopy (CLSM) images of R36A and R36A-CSP2 biofilms. R36A and R36A-CSP2 were grown for 24 h at 37°C and examined by CLSM. A representative image is shown. Live cells are stained in green and dead cells in red. Images show that R36A forms biofilms that are around 30% thicker and more densely packed than R36A-CSP2 biofilms.

The architecture of the biofilms formed by R36A and its isogenic CSP2 variant was evaluated by confocal laser scanning microscopy (CLSM). As can be seen in Figure 3, the wild-type strain (CSP1) forms biofilms that are thicker and more densely packed than R36A-CSP2 biofilms. These data are consistent with the differences observed in terms of CFUs described above.

### Pherotype influences pneumococcal transformation in planktonic growth

**Figure 4:**
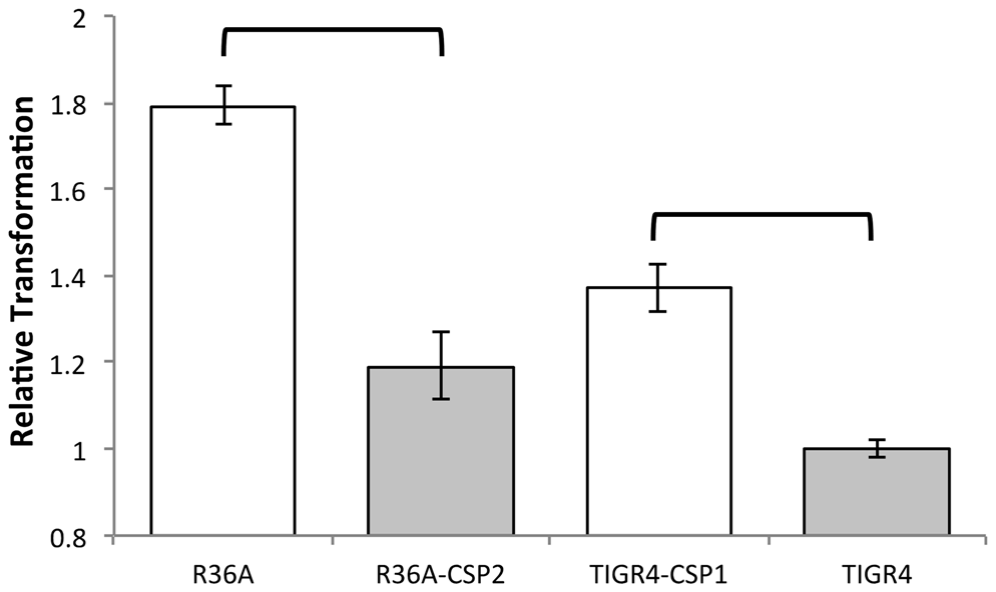
Transformation of R36A and TIGR4 and their pherotype switched derivatives in liquid culture. DNA from strain CP1500 (1) was used for transformation and transformants with streptomycin and novobiocin resistance phenotypes were screened for both pherotypes and no differences were noted. Results are a total of the observed transformants resistant to any of the two antibiotics, subtracting transformants with double antibiotic resistance, normalized by the average result obtained for TIGR4 strain (3.6×10^6^ CFU/ml). Strains harboring the *comC1* allele yield on average 1.4× more transformants than those harboring *comC2*, independently of the genetic background. Error bars represent the 95% confidence interval for the mean of 9 replicate experiments. Horizontal brackets identify groups with significantly different means (independent samples t-test, p<10^−4^).

We wanted to test if the observed differences in the capacity to form biofilms of each pherotype would also be reflected in transformation efficiency in different growth conditions since CSP is the signal triggering the competence activating cascade. We started by testing transformation of R36A, R36A-CSP2, TIGR4 and TIGR4-CSP1 in liquid culture (Figure 4). The strains carrying the *comC1* allele yielded 50% and 37% (for R36A and TIGR4, respectively) more transformants than their isogenic *comC2* strains (Student's *t* test, p<10^−4^ for both R36A and TIGR4 genetic backgrounds). A similar result was obtained previously when transformation in liquid culture of both *comC1* and *comC2* strains was compared in strains where the *comC* locus was deleted and competence was induced by artificial CSP addition [38]. The experiments reported were performed in a genetic background similar to R36A and the *comC1* strain yielded 56% more transformants than the *comC2* strain, a comparable result to ours.

### ComD binding to ComC is influenced by pherotypes

In order to test for the binding capacity of both ComD1 and ComD2 to their cognates peptides, we started by constructing knock-out mutants of the *comC* gene: R36AΔ*comC*, TIGR4-CSP1Δ*comC*, R36A-CSP2Δ*comC* and TIGR4Δ*comC*. All these mutants were able to sense external CSP and to induce the competence signalling cascade, but were not able to produce their own CSP molecules. By using this experimental design we were able to control the exact concentrations of CSP that cells were exposed to, without the bias of self-produced CSP. Also, due to the fact that we used pairs of isogenic mutants that differed only in the ComD receptor, the resulting differences in the transformation efficiency in response to increasing external CSP concentrations can be attributed only to differences in ComD/CSP binding.

**Figure 5:**
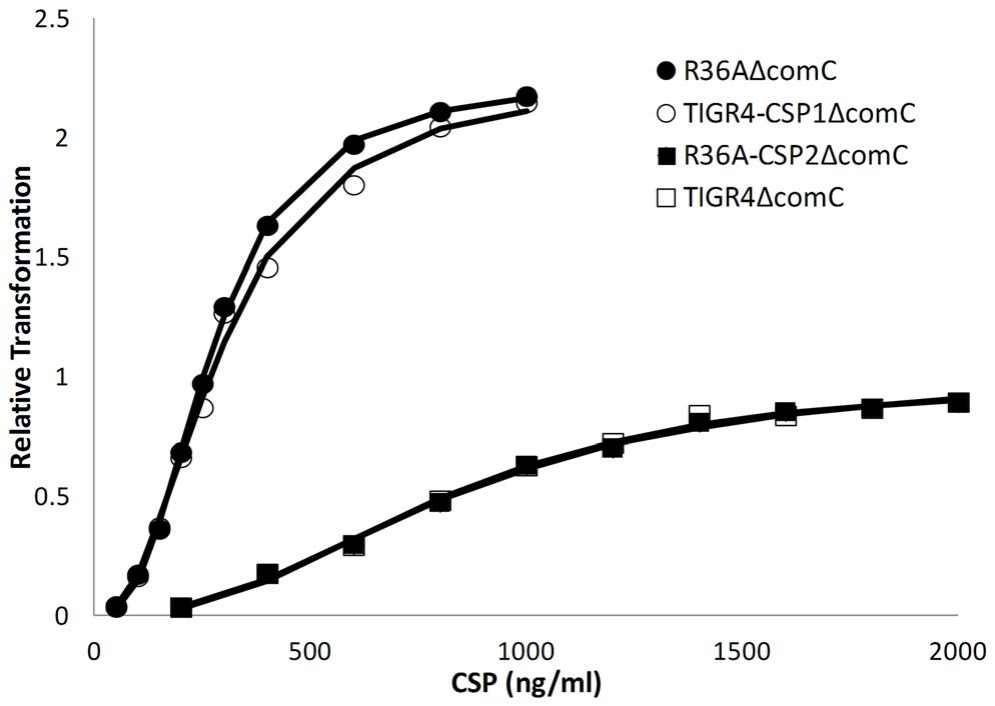
Comparison of the transformation efficiency of Δ*comC* strains in response to external CSP. Mutant strains were incubated with donor DNA and a gradient of their cognate CSP. Transformants resulting from each added concentration were counted as a measure of transformation efficiency. Results show that strains with ComD1 receptor present higher numbers of transformants and need less external CSP to reach the same transformation levels as their CSP2 counterparts. Plotted values are an average of 3 independent replicates and were normalized to the maximal transformation efficiency of TIGR4Δ*comC* strain. Lines represent adjusted Hill functions, obtained through non-linear least-squares regression.

Results show that the relationship between transformation efficiency and CSP concentration present a sigmoidal behavior, typical of cooperative phenomena (Figure 5). The initial binding of the peptide to its receptor induces the expression of ComD which will facilite the binding of more peptide to the cell. This positive cooperativity can be responsible for the observed sigmoidal curves. These curves are well-fitted by Hill functions, characterized by the maximal transformation efficiency value (*T*), by the CSP concentration needed to reach half-maximal transformation efficiency (*K_1/2_*) and the Hill coefficient (*n*). The parameter values resulting from the adjustment of Hill functions to the observed data for the four strains are presented in table 1. Strains with the same ComD receptor but in different genetic backgrounds show overlapping transformation efficiency curves. All four strains have similar Hill coefficients, as expected since the process is essentially the same in the four strains. Both strains with ComD1 show higher *T* values and lower K_1/2_ values when compared to ComD2 strains. The latter differences probably reflect a higher binding affinity of the CSP1 peptide to the ComD1 receptor, leading to the competence cascade reaching higher levels of activation with lower doses of CSP. A potential mechanism could be the expression of a larger number of ComD1 receptors on the surface of individual bacteria, resulting from the positive feedback of CSP in the expression of its receptor and the higher activating-capacity of the competence cascade by the CSP1 pheromone.

**Table 1:** Hill function parameters estimated by non-linear regression for the transformation efficiency assay of Δ*comC* strains.

Mutant strain	*T* (CI 95%)*	*K_1/2_ ng/ml* (CI 95%)	*n* (CI 95%)
**R36AΔ** ***comC***	2.24 (2.19; 2.29)	272 (264; 281)	2.63(2.45; 2.82)
**TIGR4-CSP1Δ** ***comC***	2.25 (2.05; 2.44)	295 (260; 329)	2.27 (1.80; 2.73)
**R36A-CSP2Δ** ***comC***	1.02 (0.93; 1.10)	832 (751; 913)	2.39 (1.99; 2.79)
**TIGR4Δ** ***comC***	1.00 (0.90; 1.10)	809 (718; 900)	2.50 (1.96; 3.03)

* *T* were normalized by the maximal transformation efficiency obtained for TIGR4Δ*comC* (10^7.62^ CFU/ml).

### Intra-pherotype recombination prevails within biofilms

**Figure 6:**
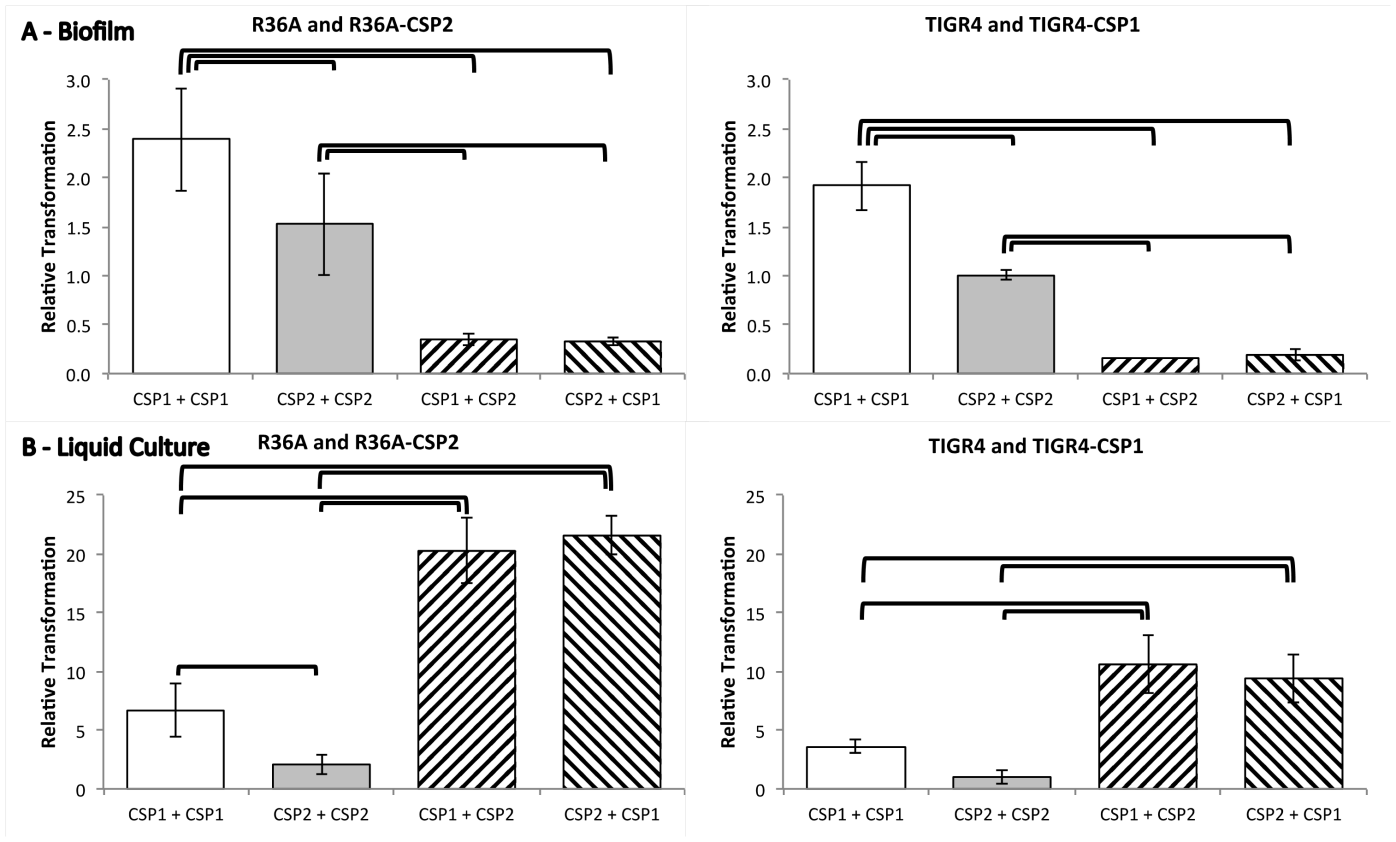
Comparison of intra-pherotype and inter-pherotype recombination in biofilms and liquid cultures. Each bar represents the fraction of double resistant CFUs in the total number of CFUs, normalized by the same fraction obtained for TIGR4 intrapherotype transformation (0.048 in biofilm and 4.1×10^−6^ in liquid culture). Experiments were performed both in biofilm (panel A) and in liquid culture (panel B) by mixing equal amounts of two strains, each with one antibiotic resistance marker (novobiocin and streptomycin). In each experiment, the two leftmost bars represent mixtures of strains with the same pherotype and on the right pherotypes were mixed to evaluate inter-pherotype recombination. White bars represent mixtures of CSP1 producing strains, grey bars CSP2 producing strains and hatched bars mixtures of CSP1 and CSP2 producing strains. In the left slanted hatched bars the CSP1 producing strain carried the novobiocin resistance marker, while the CSP2 producing strain carried the streptomycin resistance marker. In the right slanted hatched bars the resistance markers on the strains were reversed. Results show that in biofilms intra-pherotype recombination is more frequent than inter-pherotype recombination. The opposite relationship is observed in liquid culture. Error bars represent the 95% confidence intervals of the mean of 3 or 9 (biofilms and liquid culture, respectively) independent experiments. Each independent biofilm experiment consisted of 3 technical replicates. ANOVA was applied to identify significant differences in the fraction of transformants between the four types of mixed cultures. Significant differences were found (p<10^−4^) for both R36A and TIGR4 backgrounds and in both biofilm and liquid culture. Groups with significant differences according to post-hoc analysis are identified in the figure by horizontal brackets. In all cases p<10^−3^, except for the difference in transformants obtained in liquid culture with the R36A background between CSP1+CSP1 and CSP2+CSP2 mixtures, where p<10^−2^.

We then performed experiments to explore intra- and inter-pherotype recombination in both biofilm and liquid culture without exogenously added DNA (Figure 6). Analysis of variance indicates significant differences between the mean proportion of doubly resistant bacteria (transformants) in biofilms (p<10^−4^ for both R36A and TIGR4 genetic backgrounds) and in liquid culture (p<10^−4^ for both R36A and TIGR4 genetic backgrounds). *Post-hoc* analysis reveals that the proportion of transformants is higher when co-culturing two R36A (CSP1) strains carrying distinct resistance markers when compared with co-culturing R36A-CSP2 strains in both biofilm (p<10^−3^) and liquid culture (p<10^−2^). The same is observed for the TIGR4-CSP1 and TIGR4 (CSP2) comparison in biofilms (p<10^−3^), but statistical significance is not reached in liquid culture. The higher transformation efficiency of CSP1 strains, already detected in liquid culture, was also observed in biofilms (Figure 6, panel A). Mixed cultures of otherwise isogenic CSP1 and CSP2 strains yield a different number of transformants than a mixture of strains producing the same CSP, in both biofilm and liquid culture and independently of the genetic background (p<10^−3^ in all four cases). Since mixtures of CSP1 and CSP2 strains with reversed antibiotic resistance markers do not present statistically different proportions of transformants, this effect cannot be attributed to a different behavior of the two antibiotic resistance markers used (Figure 5, hatched bars). However, the observed differences have opposite signs in biofilm and in liquid culture. In the latter, inter-pherotype transformation is more efficient and that can be interpreted as a consequence of a directional fratricide effect in the sense that one of the pherotypes is preferentially lysed during fratricide [39], [40]. CSP1 and CSP2 strains can reach the competent state at different times, due to the lack of response of the ComD1 and ComD2 receptors towards their heterologous CSPs [14], [15], [38]. The first pherotype to achieve competence can then induce the lysis of bacteria with the other pherotype. Limited lysis of bacteria with the same pherotype may occur, but we expect this to happen with lower frequency, since competent cells activate the expression of a lysis immunity protein ComM [41] and therefore the majority of non-competent cells will carry the opposite *comC* allele. While in an homogeneous bacterial culture only the small fraction of non-competent cells are susceptible to lysis, in mixtures of CSP1 and CSP2 strains in liquid culture the entire population carrying the *comC* allele that “lost the competence race” will be susceptible to lysis. Our data are compatible with this hypothesis, resulting in more bacterial lysis and higher amounts of free DNA for subsequent recombination.

In contrast to planktonic growth, in biofilms intra-pherotype recombination is more frequent than inter-pherotype recombination. The lower number of inter-pherotype transformants in biofilms could be biased by a different representation of each pherotype in the mixed strain biofilm. Different frequencies of each pherotype in the final biofilm population could be due to different biofilm growth rates or to a directional fratricide effect. Considering the separate biofilm growth of each pherotype, we estimated a proportion of 68.5% and 70.9% of bacteria bearing CSP1 (for R36A and TIGR4 genetic backgrounds, respectively) in the mixed biofilm in the absence of directional fratricide or other interference in biofilm growth rate due to the presence of bacteria with the CSP2 pherotype. To compare these values with what was happening in our experiments, 48 randomly picked colonies representing the population constituting a biofilm with 24 h of growth that had been seeded with CSP1 and CSP2 strains, were pherotyped. We observed proportions of colonies of the CSP1 pherotype of 72.9% (CI 95% 60.3–85.5%) and 62.5% (CI 95% 48.8–76.2%) (for R36A and TIGR4 genetic backgrounds, respectively). These are not significantly different from the predicted values, indicating that the difference in biofilm growth capacity of CSP1 and CSP2 strains could explain the frequency imbalance between the two pherotypes in the final bacterial population forming the mixed biofilm.

Considering that transformation can occur randomly between any two bacteria, with one acting as DNA donor and the other as acceptor independently of pherotype, the measured proportion of doubly resistant bacteria would be maximal if half of the total bacteria had a different antibiotic resistance marker. If one marker is more frequent than the other, more transformation events will occur between bacteria with the same marker resulting in fewer doubly resistant transformants. Accordingly, we used the expected frequencies of each pherotype and estimated that a reduction of 13.6% (95% CI 10.8–16.4%) for the R36A and 17.5% (95% CI 11.9–23.2%) for the TIGR4 genetic backgrounds, respectively, in the proportion of doubly resistant transformants can be attributed solely to the frequency imbalance of the pherotypes in the mixed biofilm. However, when we compared the proportion of doubly resistant transformants obtained in intra-pherotype or inter-pherotype transformation in biofilms, we observed reductions of 77 to 90%. This means that the lower inter-pherotype transformation efficiency is not solely due to the pherotype frequency imbalance. In other words, there is an effective reduction in the probability of inter-pherotype transformation within biofilms.

We also tested if transformation was as likely to occur with CSP1 or CSP2 bacteria acting as DNA acceptors. For that purpose, 48 doubly resistant colonies (transformants) obtained after 24 h of growth of a biofilm seeded with CSP1 and CSP2 strains, were randomly picked and pherotyped. We observed that these transformants were enriched in the CSP1 pherotype: 91% (95% CI 83.8–99.5%) of CSP1 bacteria for mixed biofilms of strains with the R36A genetic background and 83.3% (95% CI 72.8–93.9%) for mixed biofilms of strains with the TIGR4 genetic background. The proportions of CSP1 bacteria among transformants are significantly higher than those found among all bacteria in the biofilm (Fisher exact test, p<0.05 for both R36A and TIGR4 genetic backgrounds). These data are consistent with the higher transformation efficiency of isogenic CSP1 strains when compared with CSP2 strains and are compatible with limited localized directional fratricide, as documented in a larger scale in the case of planktonic growth.

Globally, these results suggest that, within biofilms, the inter-pherotype fratricide phenomenon is less frequent, potentially due to a restricted spatial organization of bacteria with different pherotypes. Spatial restrictions may impact on DNA diffusion within the biofilm. Moreover, since it is also believed that physical contact between competent and non-competent cells is required to induce lysis [41] the biofilm structure may hamper the capacity of the highly competent CSP1 bacteria inducing the lysis of their CSP2 counterparts.

## Discussion

Changes in the CSP and its receptor can potentially alter the efficiency of the competence activation pathway. An important end point of this activation is the induced lysis of a fraction of the pneumococcal population, a phenomenon known as fratricide [10], [13]. This targeted lysis is essential for the release of DNA to the environment, where it can participate in transformation events. Additionally, as it has been recently proposed in *S. pneumoniae* [29], [42] and in close related species [43], [44], released DNA can be incorporated into the biofilm extracellular matrix, enhancing biofilm development.

Comparison of isogenic CSP1 and CSP2 strains resulted in three observations supporting the existence of differences in competence of the two pherotypes and of their impact on biofilm growth: 1) transformation of otherwise isogenic CSP2 strains with exogenously provided DNA in liquid medium is less efficient than that of CSP1 strains, possibly due to a lower affinity of ComD2 for its cognate CSP binding, 2) biofilm growth of CSP2 strains is hampered relatively to CSP1 strains, 3) addition of synthetic cognate CSP amplifies the differences in biofilm growth between pherotypes.

Transformation frequency is known to vary among pneumococcal strains [15], [45]. Some of this variability can be due to variation in the *comCDE* locus, particularly in the CSP receptor ComD that presents several alleles [45]. Furthermore, it is not surprising that even among strains expressing the same CSP1 allele transformation can vary greatly since numerous genes are induced upon competence [11] and these can impact on the overall efficiency of transformation. Supporting this view, our data show that the transformation efficiency of strains with the TIGR4 genetic background was always lower than that of R36A strains with the same pherotype. Perhaps the most extreme example is the existence of non-transformable strains due to inactivation of critical genes downstream of the triggering signal, such as the competence pilus structural gene *comYC* [46]. Moreover, we show that in two distinct genetic backgrounds, isogenic strains carrying the *comC*2 allele present a consistent reduction in transformation efficiency relative to strains carrying the *comC1* allele, in both planktonic and biofilm contexts. In other words, although the effect of producing different CSPs will certainly be modulated by variation in other important genes for competence, the data presented argues that everything else being equal, the nature of the signal triggering the competence cascade influences the extent to which DNA is exchanged by transformation. Given the multiple pathways that can be modulated by the competence induced sigma factor ComX, it will be interesting to determine what differences if any in global transcription profiles result from changing the CSP alleles.

Biofilms may be the preferred lifestyle of bacteria during asymptomatic colonization of the nasopharynx [4], [5] and competence triggered fratricide has been implicated in providing eDNA for incorporation in the biofilm extracellular matrix [29], [47]. The higher capacity of CSP1 strains to develop biofilms can then lead to higher transmissibility or longer duration of colonization. Both effects can help explain the higher prevalence of the CSP1 pherotype in pneumococcal populations [16], [21].

Unlike when causing infections, multiple pneumococcal strains simultaneously colonizing one individual may be the norm [17]–[19]. Co-colonization offers the opportunity for gene exchange that can occur through several mechanisms such as conjugation, transduction or, particularly in the case of pneumococci, through competence mediated transformation. The later is considered the major mechanism of generating genetic diversity in this species, driving the evolution of *Streptococcus pneumoniae* [48]. Exchange of DNA within biofilms may be the dominant context in which transformation occurs *in vivo* and the importance of fratricide for this transfer to occur was already shown [49]. As seen here, it was also previously noted that transformation efficiency in biofilms is higher than in planktonic growth, possibly due to multiple factors including the availability of DNA in the biofilm extracellular matrix, capsule downregulation and successive waves of competent cells arising during biofilm growth [47]. Natural nasopharyngeal colonization with strains of different pherotypes was found to be dictated by the overall frequency of the pherotypes in the colonizing bacterial population [17], [18], indicating that pherotypes do not restrict co-colonization. This is in agreement with our data showing that dual-strain biofilms can be obtained with strains of different pherotypes. But we also showed that different pherotypes influenced transformation frequency within biofilms, an effect that could impact on the genetic exchange occurring during colonization.

In a recent population genetic study, we suggested that a predominance of intra-pherotype recombination is driving genetic differentiation between pneumococcal strains with different pherotypes [16]. The results presented here clarify the apparent conflict between a higher inter-pherotype transformation efficiency due to fratricide suggested previously from planktonic growth experiments [41] and the results of the population study. The data reported in this paper supports the population study indicating that genetic exchange between strains with different pherotypes was less frequent than intra-pherotype gene exchange within biofilms, which is likely to be associated with colonization where most genetic exchange is expected to occur.

By showing that pherotypes differ both in their biofilm formation and transformation efficiency, with a clear advantage of CSP1 strains, we provide an intriguing example of the possibly broad impact of limited genetic differences (in this case restricted to the *comCDE* operon) on the bacterial phenotype that may be reflected in host-pathogen interactions and on the population biology of a given species.
